# Electrospun Nanofibrous Membranes Based on Citric Acid-Functionalized Chitosan Containing rGO-TEPA with Potential Application in Wound Dressings

**DOI:** 10.3390/polym14020294

**Published:** 2022-01-12

**Authors:** Elena Cojocaru, Jana Ghitman, Gratiela Gradisteanu Pircalabioru, Cristina Stavarache, Andrada Serafim, Eugeniu Vasile, Horia Iovu

**Affiliations:** 1 Advanced Polymer Materials Group, University Politehnica of Bucharest, 1-7 Gh. Polizu Street, 011061 Bucharest, Romania; elena.cojocaru3105@upb.ro (E.C.); jana.ghitman@upb.ro (J.G.); cristina.stavarache@upb.ro (C.S.); andrada.serafim0810@upb.ro (A.S.); 2 Research Institute of the University of Bucharest (ICUB), University of Bucharest, 91-95 Splaiul Independentei, 050095 Bucharest, Romania; gratiela.gradisteanu@icub.unibuc.ro; 3 “C. D. Nenitescu” Institute of Organic Chemistry, 202-B Splaiul Independentei, 060023 Bucharest, Romania; 4 Department of Science and Engineering of Oxide Materials and Nanomaterials, Faculty of Applied Chemistry and Material Science, University Politehnica of Bucharest, 1-7 Gh. Polizu Street, 011061 Bucharest, Romania; eugeniuvasile@yahoo.com; 5 Academy of Romanian Scientists, 54 Splaiul Independentei, 050094 Bucharest, Romania

**Keywords:** citric acid-functionalized chitosan, rGO-TEPA, nanofibrous architecture, in vitro cytocompatibility, anti-biofilm activity

## Abstract

The present research work is focused on the design and investigation of electrospun composite membranes based on citric acid-functionalized chitosan (CsA) containing reduced graphene oxide-tetraethylene pentamine (CsA/rGO-TEPA) as materials with opportune bio-properties for applications in wound dressings. The covalent functionalization of chitosan (CS) with citric acid (CA) was achieved through the EDC/NHS coupling system and was checked by ^1^H-NMR spectroscopy and FTIR spectrometry. The mixtures to be electrospun were formulated by adding three concentrations of rGO-TEPA into the 1/1 (*w*/*w*) CsA/poly (ethylene oxide) (PEO) solution. The effect of rGO-TEPA concentration on the morphology, wettability, thermal stability, cytocompatibility, cytotoxicity, and anti-biofilm activity of the nanofibrous membranes was extensively investigated. FTIR and Raman results confirmed the covalent and non-covalent interactions that appeared between the system’s compounds, and the exfoliation of rGO-TEPA sheets within the CsA in the presence of PEO (CsA/P) polymer matrix, respectively. SEM analysis emphasized the nanofibrous architecture of membranes and the presence of rGO-TEPA sheets entrapped into the CsA nanofiber structure. The MTT cellular viability assay showed a good cytocompatibility with the highest level of cell development and proliferation registered for the CsA/P composite nanofibrous membrane with 0.250 wt.% rGO-TEPA. The designed nanofibrous membranes could have potential applications in wound dressings, given that they showed a good anti-biofilm activity against Gram-negative *Pseudomonas aeruginosa* and Gram-positive *Staphylococcus aureus* bacterial strains.

## 1. Introduction

Wound dressings represent a modern, versatile, and accessible therapeutic approach, used in order to ensure wound protection against infections and to promote the healing process [1]. For designing an ideal wound dressing, certain features must be considered, such as biocompatibility, high absorbency for wound exudates, good permeability for oxygen and water vapor to accelerate wound healing, and relevant antibacterial activity [2].

The antibacterial activity of chitosan (CS) is provided by the presence of protonated amino (-NH_2_) groups in acidic medium and plentiful hydroxyl (-OH) functionalities [3]. Furthermore, this biopolymer exhibits several essential characteristics which make it an appropriate material for applications in the wound dressings field, such as hemostatic activity, muco-adhesion, biocompatibility, and biodegradability, as well as low toxicity [4]. Additionally, both hemostatic activity and muco-adhesion properties are related to the polycationic character given by the presence of positive charges [5], which allow CS to interact with a variety of negatively charged compounds.

**Figure 1:**
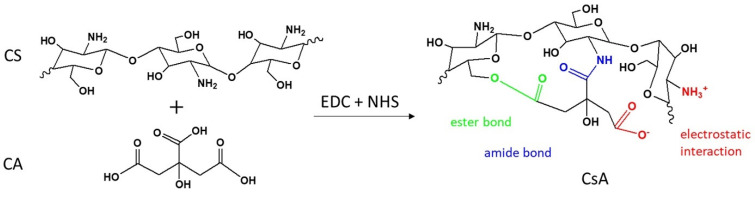
The CS covalent functionalization with CA using the EDC/NHS coupling system.

The literature reports a great number of studies which describe various strategies in order to improve the antibacterial properties and water solubility of CS [6]. The functionalization of CS with certain compounds that possess an additional number of hydrophilic functional groups, such as -OH and carboxyl (-COOH), may be an alternative. One of these compounds is citric acid (CA), a ternary carboxylic acid with high hydrophilicity and extensive applications in the food industry, chemical engineering, and medicine due to its good antibacterial activity [7]. The main roles of CA consist in maintaining the hydrophilic balance of the materials and the involvement in hydrogen bonding interactions with other biomolecules due to the increased number of -COOH functional groups [8]. Therefore, the negative charges of CA preponderantly originated from -COOH groups facilitate the cooperation with positive functionalities of CS in several ways: (1) interactions between -NH_2_ of CS and -COOH of CA, forming the amide bonds, (2) interactions between -OH of CS and -COOH of CA, generating ester bonds, and (3) electrostatic interactions between ammonium (NH_3_^+^) with a positive charge of CS and carboxylate (COO^−^) with a negative charge of CA [9], as shown in Figure 1.

The CS covalent functionalization with CA was performed using the 1-Ethyl-3-(3-dimethyl amino-propyl) carbodiimide (EDC)/N-hydroxy-succinimide (NHS) coupling system. EDC works by activating the -COOH groups from the CA structure, in acidic conditions, to form the amine-reactive O-acyl-isourea intermediate, which is unstable in aqueous solutions. In the presence of NHS, its stability is enhanced, allowing the efficient coupling with -NH_2_ groups of CS via amide bonds, leading to the formation of CA-functionalized CS (CsA) and the water-soluble isourea as by-products [10,11,12].

Electrospinning represents a modern and widely used technology to generate thin fibers with diameters in the nanometer range with controlled architecture and different configurations by varying certain solution or process parameters and the electrospinning system/mode [13,14].

The design of electrospun nanofibers based on CsA is problematic due to the polycationic character and high viscosity of CS [15]. In order to enhance the electrospinnability properties of CsA solution, synthetic polyethylene oxide (PEO) with high molecular weight may be used.

So far, the literature reports few research studies in which the functionalization of CS with CA to formulate materials with advanced and suitable characteristics for a particular biomedical application is approached. For instance, Chen and co-workers developed the drug-loaded CA-modified CS hydrogels with application in wound dressing [16]. Xu’s study reported the synthesis of CS beads functionalized with CA soaked with human hair fiber with high lysozyme adsorption capacity [17]. In another study, Rajan formulated cisplatin-loaded CS-based nanocomposites crosslinked with poly-oxalates and CA as potential carriers for controlled and targeted anticancer therapy [18]. Bagheri showed that the CA-functionalized CS nanoparticles have an adsorption potential to remove Cr (VI) from contaminated wastewaters [19], while Safaei-Ghomi demonstrated the catalytic efficiency of CS functionalized by CA in organic synthesis of benzo-pyrano-pyridines by a multicomponent reaction [20].

Reduced graphene oxide (rGO) is achieved by chemical reduction of oxygen-containing functionalities (-OH, -COOH, carbonyl -C=O) on the surface of GO nanosheets in the presence of diverse reducing agents [21,22]. The rGO can be of several types, such as amine-functionalized rGO (rGO-NH_2_) and piperazine-functionalized rGO (rGO-NH), including tetraethylene pentamine-functionalized rGO (rGO-TEPA) which presents enhanced stability due to the covalent binding of TEPA to the rGO surface [23]. Recent investigations revealed that rGO-TEPA can be preponderantly used in the biosensors field due to the large specific surface area and high electrical conductivity. Cao and colleagues designed an electrochemical biosensor based on 3D paper for quantifiable determination of glucose [24]. Then, the same research team developed a sensitive electrochemical immunosensor based on nanoparticles co-impregnated with rGO-TEPA to identify the carcinoembryonic antigen [25]. In another research study, Zhang and co-workers employed rGO-TEPA and bimetallic gold–silver nanoclusters to design a non-enzymatic immunosensor with high sensitivity and selectivity to squamous cell carcinoma antigen [26].

In our previous research, we designed chitosan/carboxylated graphene oxide (CS/GO-COOH) composite scaffolds with nanofibrous architecture, using three concentrations of GO-COOH. From structural and morphological analyses, we observed the uniform distribution of GO-COOH sheets along the fibers and also a minor tendency of GO-COOH sheets to agglomerate at higher concentrations. The in vitro biological experiments (MTT, LDH) showed a good cell proliferation potential of CS/GO-COOH composite scaffolds, which was slightly affected by the presence of GO-COOH agglomerates [27].

To the best of our knowledge, composite electrospun membranes based on CA-functionalized CS in combination with rGO-TEPA have not yet been reported in the literature. The main purpose of this research was to design reduced graphene oxide tetraethylene pentamine (rGO-TEPA)-containing citric acid-functionalized chitosan (CsA) composite membranes with nanofibrous architecture as promising biomaterials with potential applications in the wound dressing field. The CsA/rGO-TEPA composite membranes with various content of rGO-TEPA were engineered using the electrospinning technique and further were chemically stabilized through crosslinking treatment using glutaraldehyde (GA) vapors. By dispersion of rGO-TEPA within the CsA/PEO polymer blend, it is expected to obtain a nanofibrous composite biomaterial with increased physical and thermal stability as well as improved antibacterial properties.

The obtained CsA/PEO and CsA/rGO-TEPA composite nanofibrous membranes were comprehensively studied using various characterization methods: FTIR spectrometry and Raman spectrometry were employed for structural characterization, morphological examination was performed using SEM microscopy, the hydrophilicity was assessed through water contact angle measurements, the thermal behavior was investigated by DSC analysis, and the MTT and LDH assays were used for the assessment of cellular viability and cytotoxicity of the engineered electrospun membranes, while their anti-biofilm potential was tested against *P. aeruginosa* and *S. aureus* bacterial strains.

## 2. Materials and Methods

### 2.1. Materials

Chitosan (CS) with medium molecular weight (Mw) and 75–85% deacetylation degree, citric acid (CA, 99.5%), acetic acid (99.8–100.5%), N-(3-dimethylaminopropyl)-N’-ethyl-carbodiimide hydrochloride (EDC, ≥98%), N-hydroxy-succinimide (NHS, 98%), polyethylene oxide (PEO) with a Mw of 600,000 Da, glutaraldehyde (GA) grade I, 50% aqueous solution, and dialysis bags with the mean flat width of 25 mm (MWCO 12,000–14,000 Da) were provided by Sigma-Aldrich (Sigma-Aldrich Chemie GmbH, Steinheim, Germany). Commercial reduced graphene oxide-tetraethylene pentamine (rGO-TEPA) with a concentration of 1.1 mmol NH_2_/g was supplied by NanoInnova Technologies (NIT, Toledo, Spain). The ultra-pure water was produced by Milli-Q Plus system (Millipore, MA, USA) and was used in the preparation of all solutions and in subsequent investigations.

### 2.2. Synthesis of CsA

The covalent attachment of CA to the CS polymer chain was performed using the EDC/NHS coupling system. Briefly, 0.61 g of CA solubilized in 25 mL of ultra-pure H_2_O/HCl with a pH ≈ 4.5 [28] was activated and stabilized with EDC (1.56 mmol) and NHS (0.61 mmol), respectively, as coupling agents. Afterwards, 0.61 g of CS powder was incorporated into the solution. The reaction was performed under magnetic stirring at room temperature for 72 h. The resulted solution was purified using a dialysis bag against ultra-pure water for 24 h, then the solution was casted into a Petri dish, frozen at −20 °C for 24 h, and lyophilized at a pressure of 0.005 mbar for 24 h, using the freeze-dryer Alpha 2–4 LSCbasic (Martin Christ GmbH, Steinheim, Germany). The lyophilized CsA was used in further experiments.

### 2.3. Formulation of the Electrospinning Systems

The CsA solution was obtained by solubilizing 3% (*w*/*v*) CsA in 3 M (*v*/*v*) acetic acid aqueous solution under heating at 60 °C and magnetic stirring conditions, until complete solubilization. The PEO solution was obtained by solubilizing 5% (*w*/*v*) PEO powder in ultra-pure water, under heating at 80 °C and magnetic stirring conditions for 8 h. Subsequently, the two polymer solutions were blended in a (*w*/*w*) ratio of 1/1 for 24 h, to ensure the complete homogenization, then the resulted (CsA/P) solution was exposed to the electrospinning process in order to achieve CsA-based nanofibrous samples.

The electrospun rGO-TEPA-containing CsA-based composite membranes (CsA/PG_T_) were obtained by dispersing three different concentrations of rGO-TEPA in ultra-pure water using ultrasonication treatment for 2 h. Then, to the obtained dispersions, the calculated amounts of PEO powder and CsA solution were added, to maintain the (*w*/*w*) ratio of 1/1. The composition of the prepared nanofibrous systems is presented in Table 1.

### 2.4. Optimization of the Electrospinning Parameters

The electrospinning process was performed by means of Climate-Controlled Electrospinning equipment (IME Technologies, Waalre, The Netherlands). Then, 3 mL of each prepared mixture was charged into a syringe coupled with a tube and a metallic needle at the top, having 0.6 mm in inner diameter, through which the solution was pushed using a syringe pump. The nanofibrous membranes were deposited on the rotating collector. The tip-collector distance was 15 cm, and the applied voltage was set between 12 and 18 kV. All the samples were electrospun at a temperature of 25 °C and relative humidity between 30% and 40%.

### 2.5. Crosslinking of Nanofibrous Membranes

The crosslinking of nanofibrous membranes was carried out by placing the samples above a support in a sealed cavity containing GA aqueous solution [29]. The membranes were crosslinked in GA vapors for 3 h, then were immersed in ultra-pure water for 4 days to remove free GA and dried in an oven at 37 °C for 2 h.

### 2.6. Characterization Methods

#### 2.6.1. Structural Characterization

^1^H-NMR and ^13^C-NMR spectra were recorded on a Bruker Avance III HD 600 NMR spectrometer (Bruker Biospin, Rheinstetten, Germany) at a resonance frequency of 600.12 and 150.90 MHz, respectively. Before analysis, all the compounds were solubilized in D_2_O/HCl solution (pH = 3). The chemical shifts were indicated in parts per million (ppm) and the data were processed using TopSpin 3.5 pl 6 software. Fourier Transform Infrared (FTIR) spectra were recorded on a Bruker Vertex 70 FTIR spectrometer (Bruker, Billerica, MA, USA) using the ATR method at a resolution of 4 cm^−1^ and the wavenumber range of 600–4000 cm^−1^, performing 32 scans for each sample. Raman spectra were recorded on a Renishaw inVia Raman microscope system (Renishaw, Brno-Černovic, Czech Republic) in the range of 100–3200 cm^−1^ at the excitation laser wavelength of 473 nm, and a laser power of about 10% and 3 accumulations for 10 s were used. The laser beam was focused with the 100× microscope objective on the surface of each sample.

#### 2.6.2. Rheological Characterization

The viscosity of each precursor system was determined in the shear rate ramp 2.5 × 10^−2^–2.5 × 10^2^ s^−1^, by means of a Kinexus Pro rheometer (Malvern Panalytical Ltd., Worcestershire, UK) accessorized with a Peltier component for temperature control, using a parallel plate geometry. The samples were placed on the base plate and the top plate was lowered to a fix gap of 0.5 mm. The measurements were performed at room temperature (25 °C); to avoid dehydration of the samples, a water-lock system was used.

#### 2.6.3. Morphological Characterization

The morphological features of all un-crosslinked electrospun membranes were analyzed using Quanta Inspect F50 Scanning Electron Microscopy (SEM) (FEI, Hillsboro, OR, USA) using a field emission electron gun with 1.2 nm resolution. A small section of each electrospun mesh was covered with a thin gold layer and was subjected to SEM analysis.

#### 2.6.4. Wettability Investigations

The wettability assessment of the crosslinked electrospun membranes was carried out by the Drop Shape Analyzer–DSA100 (Krüss Scientific GmbH, Hamburg, Germany) using the sessile drop method and Young Laplace equation. After deposition of a water drop on the sample surface, the shape of the droplet with a volume of 2 μL was recorded with a CF03 digital camera for 20 s, at room temperature. The values of the static water contact angle were calculated using the Advance software and represent the average value of three measurements.

#### 2.6.5. Thermal Characterization

The differential scanning calorimetry (DSC) investigation was carried out on a Netzsch DSC 204 F1 Phoenix calorimeter (Netzsch-Gerätebau GmbH, Selb, Germany), using a heating rate of 10 °C/min, in the ±20–300 °C temperature range and a constant nitrogen flow rate of 20 mL/min. Each sample weighing 7–8 mg of nanofibrous membrane was placed in a hermetically sealed aluminum pan prior to be analyzed.

#### 2.6.6. In Vitro Cytocompatibility and Cytotoxicity

The in vitro cytocompatibility of nanofibrous membranes was studied by incubating them with NCTC fibroblasts for 2 and 6 days, respectively, in DMEM medium, including 10% fetal bovine serum, supplemented with penicillin and streptomycin. The cells were seeded in 24-well plates with a density of 1 × 10^5^/mL and incubated in an atmosphere of 5% CO_2_, then were left to adhere on the surface of CsA/P, CsA/PG_T_2, and CsA/PG_T_5 fibrous materials. The MTT test allows the quantitative evaluation of living cells in culture, with the MTT compound [3-(4,5-dimethylthiazol-2-yl)-2,5-diphenyltetrazolium bromide] being permeable to living cells. Each sample was incubated in the presence of 1 mL of MTT solution with 5% CO_2_, at 37 °C for 4 h. The metabolic activity of cells was determined by quantifying the amount of tetrazolium dye MTT reduced to isopropanol-soluble formazan crystals (purple solution) by measuring the absorbance at 550 nm using a spectrophotometer (Flex Station 3).

The cytotoxic response of nanofibrous membranes was evaluated using the lactate dehydrogenase assay (LDH), a quantitative test that indicates the number of dead cells in the culture. Cells that do not have membrane integrity release the cytoplasm with LDH content into the culture medium [30]. The culture media were blended with the components of the Tox-7-KT kit, and incubated in dark conditions for 20 min. The resulted solution can be read spectrophotometrically at 490 nm. The sample-free cell culture was used as a control. The tests were carried out in triplicate.

#### 2.6.7. Anti-Biofilm Activity

The anti-biofilm potential of nanofibrous biomaterials was assessed against Gram-positive (+) (*Staphylococcus aureus* ATCC 25923—*S. aureus*) and Gram-negative (−) (*Pseudomonas aeruginosa* ATCC 27853—*P. aeruginosa*) bacterial strains. Glycerol stock solutions were striated on Mueller Hinton Agar (MHA) in order to achieve cultures of 24 h for all studies. The growth of monospecific biofilm was evaluated at 4 h after exposure. The nanofibrous membranes to be tested were sectioned in square samples with the side of 8 mm and sterilized using UV exposure for 20 min, followed by immersion in 1 mL of microbial suspension of ~10^7^ colony forming units (CFU)/mL, and were kept in contact for 4 h. After this time, the resulted microbial suspensions were vortexed, diluted in series ten-fold, and 10 μL of each dilution was plated on nutrient agar in three exemplars. After 24 h of incubation at a temperature of 37 °C, viable cells were counted to obtain CFU/mL for each sample.

#### 2.6.8. Statistical Analysis

The results were depicted as mean values with their standard deviation (mean ± S.D.). The statistical analyses were performed using the one-way ANOVA test and the differences were considered significant if *p* < 0.05.

## 3. Results and Discussion

### 3.1. Structural Characterization of Functionalized Raw Material (CsA)

**Figure 2:**
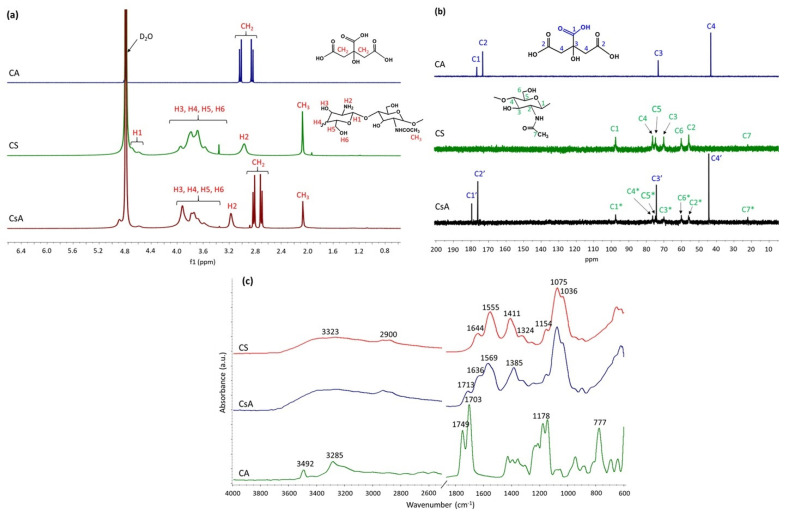
Structural characterization of CsA: (**a**) ^1^H-NMR spectra of CA, CS, and CsA in D_2_O/HCl at 25 °C. (**b**) ^13^C-NMR spectra of CA, CS, and CsA in D_2_O/HCl at 25 °C. (**c**) ATR-FTIR spectra of CA, CS, and CsA.

NMR spectroscopy is one of the most powerful and conclusive analyses used in order to highlight the covalent functionalization of CS with CA, and respectively the synthesis of the CsA final product (Figure 2a,b).

The ^1^H-NMR spectrum of CS (Figure 2a) is characterized by the following specific signals at 2.07, 2.97, 3.36–3.96, and 4.60–4.79 ppm, which can be attributed to the protons of the methyl (-CH_3_) group from N-acetyl-glucosamine related to the deacetylation degree [31]: H2 of the glucosamine unit, H3, H4, H5, and H6 of the glucopyranose moieties, and anomeric H1 [32], respectively. The peaks assigned to the -CH_3_ group from N-acetyl-glucosamine (2.07 ppm) and those corresponding to glucopyranose units from the region of 3.00–4.00 ppm are also found in the ^1^H-NMR spectrum of CsA. The ^1^H-NMR spectrum of CA exhibits a signal of doublet of doublets in the range of 2.83–3.04 ppm, corresponding to the protons of methylene (-CH_2_-) groups.

In the ^1^H-NMR spectrum of CsA, it can be observed that after the functionalization reaction, the doublet of the doublets peak was shifted towards lower values (2.69–2.83 ppm), indicating the successful covalent attachment of CA on the CS polymer chains [33]. Additionally, in the ^13^C-NMR spectrum of CsA (Figure 2b), it can be observed that besides the four carbon atoms characteristic for CA (C1′—44.37 ppm, C2′—74.29 ppm, C3′—176.11 ppm, C4′—179.59 ppm), the carbon atoms coming from the molecular structure of CS are also shown (C1*, C2*, C3*, C4*, C5*, C6*, C7*) [34].

Then, ATR-FTIR spectrometry, as a complementary technique for ^1^H-NMR spectroscopy, was used to confirm the covalent functionalization of CS with CA (the synthesis of CsA), and the recorded FTIR spectra of CS, CA, and CsA are shown in Figure 2c.

The FTIR spectrum of CS exhibits typical bands at 3323 cm^−1^ (stretching vibration of O-H and N-H bonds) and around 2900 cm^−1^ (C-H symmetric and asymmetric stretching of -CH_2_- groups). The peaks at 1644 cm^−1^ (C=O stretching of amide I), 1555 cm^−1^ (N-H bending vibration of amide II), and 1324 cm^−1^ (C-N stretching of amide III) confirmed the existence of residual N-acetyl groups [35]. The absorption peaks at 1411 and 1154 cm^−1^ are correlated with the presence of C-H bending and asymmetric stretching of the C-O-C bridge, respectively, while the bands at 1075 and 1036 cm^−1^ can be assigned to C-O bond stretching vibration [36].

The FTIR spectrum of CA is characterized by the following signals, at 3492 and 3285 cm^−1^ that indicate the stretching vibration of O-H, 1749 and 1703 cm^−1^ corresponding to C=O stretching of aliphatic carboxylic acid, 1178 cm^−1^ is ascribed to stretching vibration of C-O from C-OH, and 777 cm^−1^, which is correlated with CH_2_ rocking [37].

The appearance of a new peak at 1385 cm^−1^ in the FTIR spectrum of functionalized CsA, which may be attributed to amide III, confirmed that an amidation reaction took place between -NH_2_ groups of CS and -COOH groups of CA. Moreover, the shift towards higher values of a characteristic C=O (COOH) peak of CA, from 1703 to 1713 cm^−1^, suggests the consumption of some -COOH groups in the amidation reaction, and could also indicate that ester bonds have formed between -OH groups of CS and -COOH groups of CA [38]. In addition to these modifications, it was observed that the -OH bands (3400–3200 cm^−1^) and those corresponding to C-O bond stretching from the CS spectrum (1075 and 1154 cm^−1^) were also identified in the CsA spectrum, indicating the successful synthesis of CsA.

### 3.2. Rheological Studies of the Precursor Systems

**Figure 3:**
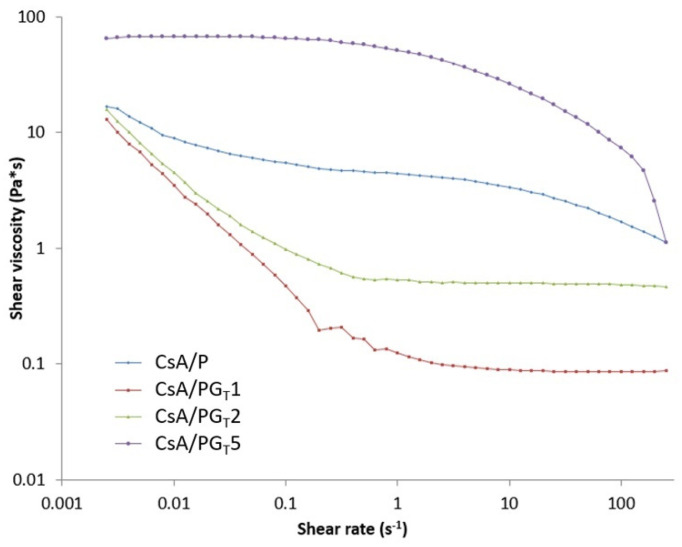
Flow curves registered for the prepared precursor systems.

The impact of rGO-TEPA on the flow rate of precursor systems was studied by rheological analyses. To this end, flow curves were registered in the interval 2.5 × 10^−2^–2.5 × 10^2^ s^−1^. The results show that all precursors exhibit a shear thinning behavior, with their viscosity decreasing with the increase of the shear rate value. However, important differences were noticed between the flow behaviors of the tested solutions. As depicted in Figure 3, any addition of rGO-TEPA in the polymer solution (CsA/P) leads to a modification of the flow behavior. In the studied shear rate interval, the shear thinning behavior of the control sample was less pronounced when compared to the composite solutions and it did not exhibit a Newtonian plateau.

The addition of low amounts of rGO-TEPA (0.125% and 0.250%, respectively) led to an obvious decrease of viscosity when compared to the control sample (CsA/P). Before reaching the Newtonian plateau, at shear rates lower than 1 s^−1^, the CsA/PG_T_1 and CsA/PG_T_2 systems exhibited a pronounced shear thinning behavior. These data indicate that the addition of low amounts of rGO-TEPA leads to local disentanglement of the polymeric chain. The CsA/PG_T_5 composition presents quite the opposite behavior when compared to the other two composite systems. The Newtonian plateau exhibited by this sample is registered at low shear rates (2.5 × 10^−2^–1 s^−1^), followed by a shear thinning behavior. These results indicate that the addition of 0.500 wt.% rGO-TEPA leads to the formation of linked structures into the polymer matrix through attractive interactions or non-covalent bonding, such as hydrogen bonds [39,40] between -COOH functionalities of CsA, ether groups of PEO, and primary and secondary amines of rGO-TEPA, that brake at shear rates higher than 1 s^−1^.

### 3.3. Structural Analyses of Nanofibrous Membranes

#### 3.3.1. ATR-FTIR Results

**Figure 4:**
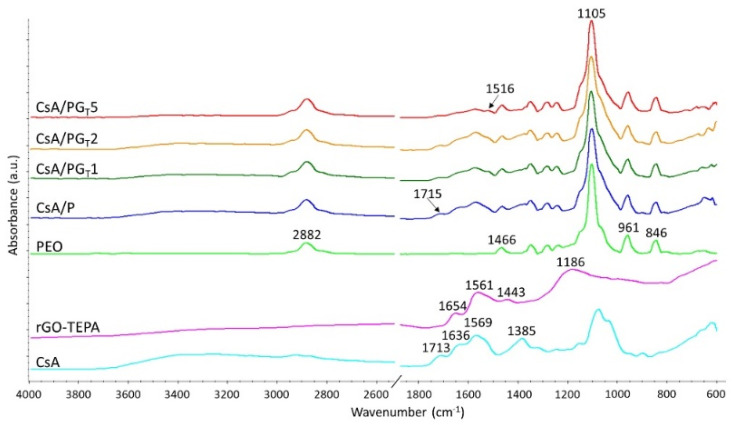
ATR-FTIR spectra of raw materials (PEO, rGO-TEPA, CsA), CsA/P, CsA/PG_T_1, CsA/PG_T_2, and CsA/PG_T_5 nanofibrous membranes.

ATR-FTIR spectrometry was used to check the chemical structure of the composite electrospun membranes as well as to assess the molecular interactions that took place between the compounds. Figure 4 shows the FTIR spectra of raw materials (PEO, rGO-TEPA, CsA) and CsA/P, CsA/PG_T_1, CsA/PG_T_2, and CsA/PG_T_5 composite nanofibrous membranes.

The FTIR spectrum of PEO exhibited characteristic peaks at 2882 and 1466 cm^−1^ assigned to the C-H asymmetric stretching vibration of the -CH_2_- group and -CH_2_- scissoring, respectively. The strong peak at 1105 cm^−1^ corresponds to C-O-C asymmetric stretching, whereas the stretching vibrations of CH_2_-CO presented in the PEO chemical structure are attributed to the peaks at 961 and 846 cm^−1^ [29].

In the FTIR spectrum of rGO-TEPA, few representative peaks were identified at 1654, 1561, 1443, and 1186 cm^−1^, which can be attributed to the C=C stretching vibrations from the planar structure of the GO sheet, N-H bending, C-N stretching vibrations, and C-C asymmetric stretching, respectively [41].

All characteristic absorption signals from both CsA and PEO chemical structure were identified in the FTIR spectra registered for electrospun CsA/P and CsA/PG_T_ composite membranes, with small shifts of the peaks’ values due to the non-covalent interactions that could appear between the constituents, such as hydrogen bonds, indicating the combination of polymers into nanofibrous membranes. Additionally, a new signal at 1516 cm^−1^ was identified in the spectra of composite nanofibrous membranes and can be attributed to the mixture of the C–N stretching of amide III with N–H bending of amide II [42] formed between –NH_2_ groups of rGO-TEPA and –COOH groups of CsA during blending.

#### 3.3.2. Raman Spectrometry Results

**Figure 5:**
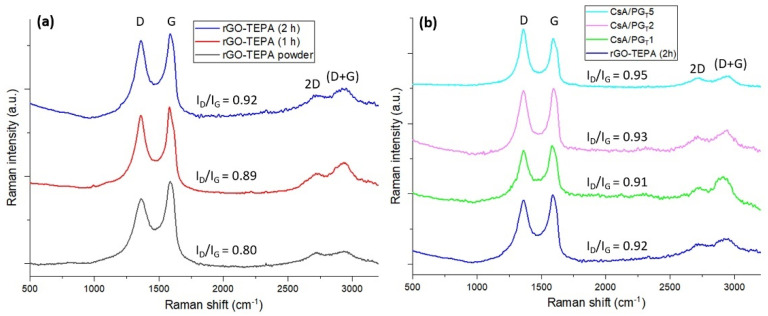
Raman spectra of (**a**) rGO-TEPA powder and after different ultrasonication times, and (**b**) CsA/PG_T_ composite nanofibrous membranes with various rGO-TEPA content.

Raman spectrometry is a valuable technique used to investigate the graphene-based materials, providing considerable information about the chemical modification of the rGO-TEPA surface. Raman spectra registered for raw material (rGO-TEPA) and ultrasonicated rGO-TEPA after 1 and 2 h (Figure 5a), as well as the Raman spectra recorded for composite nanofibrous membranes (Figure 5b), are characterized by four specific signals: D (~1350 cm^−1^), G (~1580 cm^−1^), 2D (~2700 cm^−1^), and (D + G) (~2900 cm^−1^).

The D band is associated with the occurrence of structural defects of the rGO-TEPA sheet caused by the attachment of CsA and PEO to the surface of rGO-TEPA, generating the sp^3^-carbon atoms as a result of rGO-TEPA structure modification. The G band is attributed to the stretching vibrations of sp^2^-hybridized carbon atoms from the planar structure of rGO-TEPA. The appearance of the 2D band describes the number and arrangement of rGO-TEPA layers, while the investigation of a high disorder structure of rGO-TEPA is possible through the (D + G) combination peak. The ratio between the D and G bands’ intensities (I_D_/I_G_) is applied to characterize the level of disorder in the rGO-TEPA structure and is correlated with physical processes that may have occurred in the exfoliation step [29].

Figure 5a shows the spectra of rGO-TEPA powder and rGO-TEPA registered after 1 and 2 h ultrasonication times. An increase of I_D_/I_G_ ratio values and differences regarding the shape of the 2D band and (D + G) of the raw rGO-TEPA and ultrasonicated samples can be clearly observed. Hence, the ultrasonication time influenced the I_D_/I_G_ ratio, where the longer ultrasonication time led to the increase of the I_D_/I_G_ ratio, due to the creation of more defects in the rGO-TEPA structure, according to Yang’s work [43]. Additionally, the 2D band and (D + G) combination peak from the ultrasonicated samples’ spectra are more obvious than in the raw rGO-TEPA spectrum, suggesting the presence of more exfoliated structures with a higher degree of disorders.

The values of the I_D_/I_G_ ratio of composite nanofibrous membranes calculated from the Raman spectra presented in Figure 5b were higher as compared to that of the raw material rGO-TEPA (0.92), except for the CsA/PG_T_1 sample, where a lower I_D_/I_G_ ratio value was obtained (0.91). This slight decline may indicate that the defects’ content from the rGO-TEPA structure was lower compared to the other samples due to the poor interaction between the CsA/P matrix and rGO-TEPA. When the concentrations of 0.250 wt.% and 0.500 wt.% rGO-TEPA (CsA/PG_T_2 and CsA/PG_T_5, respectively) were used, the I_D_/I_G_ ratio increased to 0.93 and 0.95, respectively, which means growth of the defects amount in the rGO-TEPA structure and the formation of sp^3^-hybridized carbon atoms due to the attachment of CsA and PEO through chemical interactions. The 2D band from the spectra of composite nanofibrous samples did not show significant differences in shape, but the (D + G) combination peak was more intense in the composite membranes’ spectra than in that of rGO-TEPA, which may suggest the random rearrangement of rGO-TEPA layers caused by the interactions between rGO-TEPA sheets and CsA/P polymer matrix [44]. The highest value of the I_D_/I_G_ ratio was registered for the CsA/PG_T_5 sample (0.95), suggesting a good exfoliation degree of rGO-TEPA within the CsA/PEO matrix, in correlation with further SEM micrographs.

### 3.4. Morphological Examination of Nanofibrous Membranes Using SEM Images

The main advantages of materials with nanofibrous architecture for applications in the biomedical field are represented by structural similarity to the extracellular medium and the fact that they provide support for cellular adhesion and proliferation on the surface of biomaterial with a large specific surface area [45].

**Figure 6:**
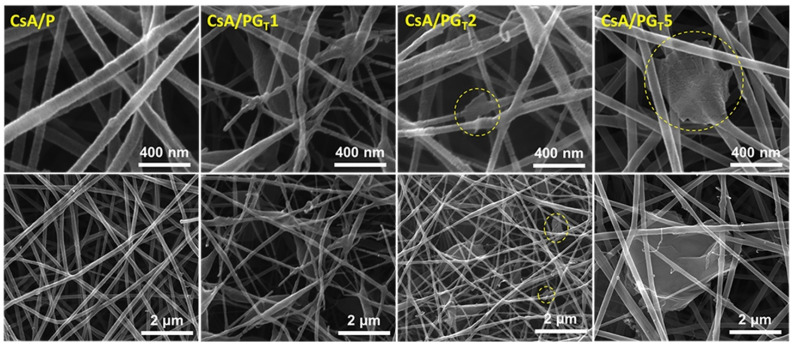
SEM micrographs of CsA/P- and rGO-TEPA-containing CsA electrospun membranes with nanofibrous architecture, at 50,000× and 200,000× magnifications.

Figure 6 depicts the SEM micrographs of the CsA/P electrospun membrane and CsA/PG_T_ composite nanofibrous membranes with different concentrations of rGO-TEPA.

The CsA/P electrospun membranes exhibited a smooth, continuous, uniform, and bead-free nanofibrous structure. The dispersion of a low amount of rGO-TEPA (0.125 wt.%) led to the formation of an unstable structure with ultrafine, discontinuous, non-uniform nanofibers and with many nanobeads and droplets, possibly due to the rupture of intra- or inter-molecular bonds, which further led to a viscosity decrease of the precursor system (as it was shown in rheology), therefore affecting the nanofibers’ morphology.

Further, raising the rGO-TEPA concentration to 0.250 wt.% and 0.500 wt.% within the materials’ composition, the nanofibrous architecture of the composite membranes was evidently improved, probably due to the increased viscosity of the solutions determined by more interactions between -OH, -NH_2_, and -COOH functional groups of CsA and primary and secondary amines of rGO-TEPA, as shown by the Raman results. In contrast to the CsA/PG_T_1 morphology, slightly thicker, continuous, uniform nanofibers with rGO-TEPA sheets entrapped into/on the nanofiber structure were observed in the case of CsA/PG_T_2 and CsA/PG_T_5 composite samples, in corroboration with Raman investigations.

### 3.5. Wettability Properties of Nanofibrous Materials

The wettability characteristics of the crosslinked CsA/P and composite CsA/PGT nanofibrous membranes’ surfaces were evaluated by water contact angle measurements, using the sessile drop image. The obtained results are presented in Table 2.

Considering the hydrophilic character of CS and PEO polymers [46] and the super-hydrophobic nature of rGO-TEPA [47] (contact angle value was 129.3° ± 0.68) given by the decrease in oxygen-containing functionalities and by the graphene planar structure, it is important to investigate the effect of the rGO-TEPA concentration upon the wettability of CsA/PG_T_ composite membranes. It was observed that increasing the rGO-TEPA concentration led to the rise of the hydrophobic nature of the investigated samples, except the CsA/PG_T_1 composite membrane, which is the most hydrophilic sample, presenting the lowest contact angle value (20.5° ± 1.03). This decrease can be due to the low degree of interaction between rGO-TEPA and the polymer matrix, as shown in Raman and rheological results. Subsequently, when a 0.250 wt.% amount of rGO-TEPA was dispersed within the CsA/P polymer mixture, the contact angle increased by approximatively 30% (to 29.9° ± 0.45), and the addition of 0.500 wt.% rGO-TEPA led to a nanofibrous membrane with a 63% higher value of the contact angle compared to the CsA/P sample.

Generally, the analyzed nanofibrous membranes exhibited a hydrophilic character, considering the contact angle values between 20.5° and 37.7°, due to the multiple oxygen-containing functionalities (-OH from CS, -COOH from CA, -O- from PEO) on the membrane’s surface, supporting the formation of hydrogen bonds with water molecules, making them suitable for biomedical purposes.

### 3.6. DSC Investigation

**Figure 7:**
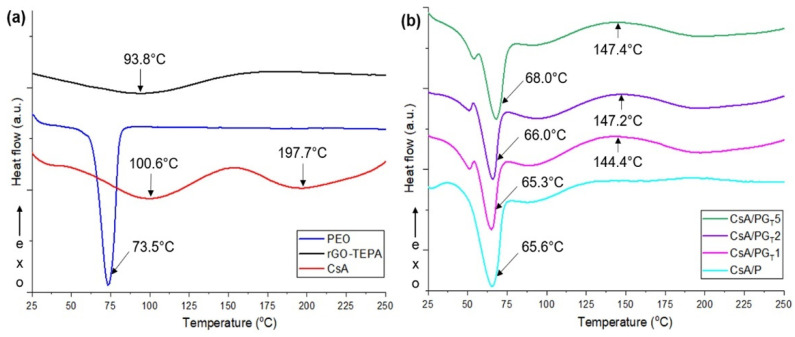
DSC curves of (**a**) raw materials, and (**b**) all investigated nanofibrous membranes.

The thermal behavior of all raw materials and engineered nanofibrous membranes with different concentrations of rGO-TEPA was assessed by DSC analysis, as shown in Figure 7a,b.

Figure 7a shows the DSC curves registered for raw materials. The thermogram of rGO-TEPA highlights a broad endothermic peak at 93.8 °C which is assigned to the dehydration process [41]. In the case of the DSC curve recorded for PEO, a strong endothermic peak located at 73.5 °C, attributed to the melting temperature (T_m_) of polymer [48], can be observed. The CsA DSC curve exhibited two endothermic peaks situated at 100.6 and 197.7 °C, which may be related to the moisture loss from the hydrophilic functionalities from the compound’s structure and the citric acid degradation, respectively, as described in the literature [49].

A slight increasing trend of the T_m_ value with the growth of rGO-TEPA concentration was observed when DSC curves of composite membranes (Figure 7b) were analyzed and compared to the CsA/P thermogram, except the CsA/PG_T_1 membranes. Moreover, the presence of a peak at around 150 °C can be detected, which could be assigned to the decomposition temperature (T_d_) of the primary and secondary amine from TEPA functionalities of rGO-TEPA [50]. This peak is shifted to higher values with the rise of rGO-TEPA content, from 144.4 °C (for CsA/PG_T_1) to 147.4 °C (for CsA/PG_T_5), which also suggests a slight improvement in the thermal stability of composite membranes.

### 3.7. In Vitro Cytocompatibility and Cytotoxicity Assessments

**Figure 8:**
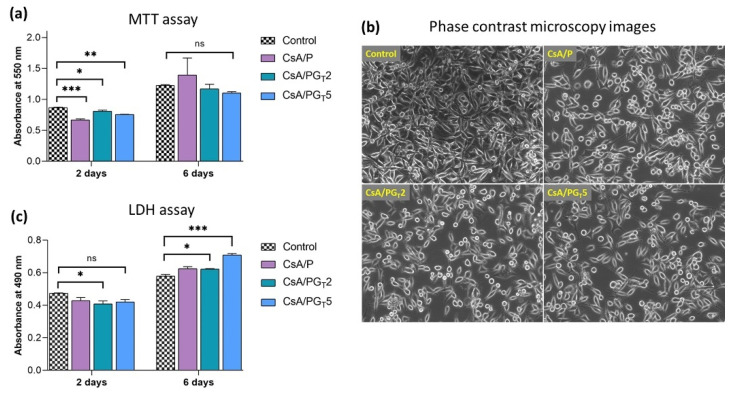
In vitro biological analysis of nanofibrous membranes. (**a**) MTT assay—viability and proliferation of NCTC fibroblast cells grown onto the surface of nanofibrous membranes, after 2 and 6 days (ns *p* > 0.5, * *p* < 0.05, ** *p* < 0.005, *** *p* < 0.0005). (**b**) Phase contrast microscopy images of NCTC cells. (**c**) LDH assay—quantification of dead NCTC fibroblasts after 2 and 6 days (ns *p* < 0.5, * *p* < 0.05, *** *p* < 0.0005).

The in vitro biological response of the designed electrospun membranes was investigated on NCTC fibroblast cells after 2 and 6 days of incubation, using MTT viability and LDH cytotoxicity assays (Figure 8). Cell culture without samples in standard conditions was used as a control.

MTT assay results (Figure 8a) demonstrated a good cytocompatibility of nanofibrous membranes after 2 days of culture. The CsA/P sample presented the lowest level of cell adhesion, whereas the CsA/PG_T_2 and CsA/PG_T_5 composite membranes showed an increase in cellular viability. The highest viability of the cells was reached by the CsA/PG_T_2 sample. According to the obtained results, after 6 days of contact with the culture medium, both the control and the electrospun membranes indicated a good cellular proliferation potential compared to the results registered after 2 days. After 6 days of culture, the composite membranes showed similar cell proliferation levels as compared to the control sample, suggesting a good degree of cytocompatibility and the possibility of composite membranes to sustain the cell growth and proliferation. Additionally, the phase contrast microscopy images of NCTC cells that proliferated on the surface of nanofibrous materials after 6 days are presented in Figure 8b. It is noted the presence of cells with elongated shape, which proliferated and grew in the monolayer adhering to the substrate [51]; however, the spherical and smaller cells unattached to the substrate were also visible in phase contrast images [52].

LDH assay results (Figure 8c) indicated that after 2 days of culture, the cytotoxicity level of all tested materials on NCTC fibroblast cells was lower than that of the control. At the same time, the nanofibrous membranes presented similar values of cytotoxicity, unlike the CsA/PG_T_2 composite membrane which exhibited the lowest cytotoxicity degree among all the analyzed samples. After 6 days, the LDH level for CsA/P and CsA/PG_T_2 composite membranes was approximately the same, but the CsA/PG_T_5 sample showed a slightly higher level of cytotoxicity, which could be due to the high concentration of rGO-TEPA [53]. It was observed that, among the investigated samples, the CsA/PG_T_2 composite membrane showed the lowest cytotoxic potential on cell culture.

### 3.8. Anti-Biofilm Activity of Nanofibrous Membranes

**Figure 9:**
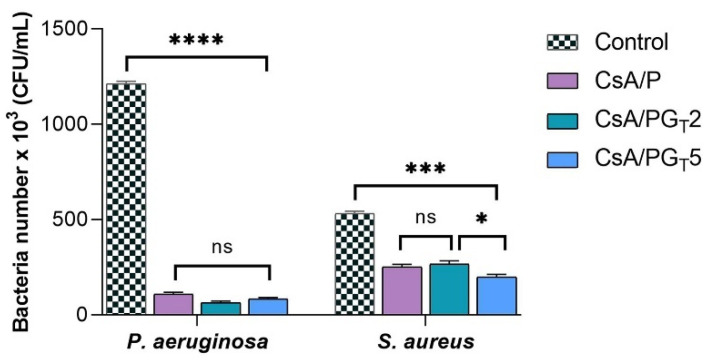
Evaluation of anti-biofilm activity of the nanofibrous membranes against *P. aeruginosa* (ns *p* < 0.5, **** *p* < 0.0001) and *S. aureus* (ns *p* < 0.5, * *p* < 0.05, *** *p* < 0.0005).

The structured multi-microbial assemblages, surrounded by a self-produced medium, which provides them protection against antibiotics and exhibits the ability to adhere to different biotic and abiotic surfaces, were identified as biofilms [54,55]. The anti-biofilm activity of nanofibrous membranes was analyzed in order to assess their capacity to inhibit the adherence of two bacterial strains (Gram (−) *P. aeruginosa* and Gram (+) *S. aureus*) in the shape of biofilm to the materials’ surface. As depicted in Figure 9, the results show that the anti-biofilm activity of nanofibrous membranes was influenced by both the composition of tested samples and the type of bacterial strain.

Considering that the obtained results are related to the number of bacteria that adhered to the materials’ surface, a higher anti-biofilm activity was clearly observed in the case of Gram (−) *P. aeruginosa* when compared with *S. aureus*, probably due to the fact that Gram (−) bacteria possess a thinner peptidoglycan layer and are coated by a lipopolysaccharide outer membrane that influences the permeability of materials. Furthermore, the high anti-biofilm activity registered for *P. aeruginosa* bacteria could be attributed to the CsA nature from the polymer matrix of nanofibrous membranes. By the functionalization of CS (positively charged) with CA, the CsA molecules became negatively charged due to the COO^−^ groups originated from CA. In the presence of Gram (−) *P. aeruginosa*, the electrostatic repulsions occur between the negatively charged cell surface and CsA with negative charges, leading to the growth inhibition of the Gram (−) bacterial strain on the surface of fibrous membranes [56]. In addition, the literature reports that the bacteria with elongated shape (Gram −) are more disturbed compared to the ones with spherical shape (Gram +) due to the larger contact surface area [57]. It was also reported that the antibacterial activity of rGO implies both physical and chemical mechanisms of action: the physical deterioration is represented by the stress of the bacterial cell membrane that is produced due to the contact with the sharp edges of rGO nanosheets, while the oxidative stress denotes a chemical action mode, through which rGO generates reactive oxygen species (ROS) that can affect the bacteria DNA, resulting in their inhibition [53]. On the other hand, the contribution of rGO-TEPA and CS to the inhibition of Gram (+) *S. aureus* bacteria is based on the electrostatic repulsion between positively charged bacterial cells and NH_3_^+^-positively charged functional groups of rGO-TEPA and CS.

The number of bacteria which adhered on the surface of investigated samples was lower in the case of both types of bacterial strains, as compared to the control sample. It can be observed that all the composite nanofibrous membranes presented a higher rate of inhibition of Gram (−) bacteria compared to Gram (+) ones. Regarding the *S. aureus* bacteria, the greatest anti-biofilm activity was shown by the CsA/PG_T_5 composite membrane, followed by CsA/P nanofibrous material and the CsA/PG_T_2 composite membrane. Therefore, all nanofibrous membranes may be considered efficient against biofilm formation in both types of bacterial strains.

## 4. Conclusions

The focus of this research study was to obtain composite rGO-TEPA-containing CsA-based electrospun membranes with nanofibrous architecture for potential application in wound dressings, using the electrospinning method. Through covalent functionalization of CS with CA, which was confirmed by ^1^H-NMR structural analysis, the system was supplemented with hydrophilic groups (-COOH), with hydrophilicity being an essential characteristic of a biomaterial that interacts with a damaged tissue.

FTIR results highlighted both covalent amide bonds that were formed after functionalization of CS with CA, and non-covalent interactions that appeared between the components of systems. Raman investigations highlighted the uniform distribution and the highest exfoliation degree of rGO-TEPA sheets within the CsA/PG_T_5 composite membrane. The formation of bead-free nanofibers observed in SEM was associated with the flow behavior of the precursor systems, also confirmed in rheological experiments. The CsA/PG_T_5 composite system generated continuous and bead-free nanofibers with fine distribution of rGO-TEPA along/into the nanofibers.

Concerning the wettability properties, all investigated crosslinked nanofibrous membranes presented a hydrophilic character, although the hydrophilicity slightly decreased with increasing the rGO-TEPA concentration. The DSC investigations denoted that the addition of rGO-TEPA slightly enhanced the thermal stability of the composite samples. The in vitro biological evaluations showed that the CsA/PG_T_2 composite membrane exhibited high cell viability and proliferation level (MTT assay), as well as the lowest cytotoxic effect on NCTC fibroblasts’ culture (LDH test). Moreover, all analyzed fibrous materials demonstrated a good anti-biofilm activity against *P. aeruginosa* and *S. aureus* bacterial strains, with higher activity against the Gram-negative bacterial strains.

Further investigations will be focused on the evaluation of the mechanical integrity, in vitro degradation, and assessment of the water vapor transmission rate of the nanofibrous membranes in order to check their applicability in the wound dressings field; afterwards, more extensive in vitro and in vivo biological investigations need to be performed to detect the rate of wound healing on a living tissue.

## Figures and Tables

**Table 1:** The composition of all electrospun nanofibrous membranes.

System	c(rGO-TEPA) wt.%
CsA/P	0
CsA/PG_T_1	0.125
CsA/PG_T_2	0.250
CsA/PG_T_5	0.500

**Table 2:** Water contact angle of all obtained nanofibrous membranes and rGO-TEPA.

Sample	Water Contact Angle (°)
CsA/P	23.2 ± 1.11
CsA/PG_T_1	20.5 ± 1.03
CsA/PG_T_2	29.9 ± 0.45
CsA/PG_T_5	37.7 ± 1.25
rGO-TEPA	129.3 ± 0.68

## Data Availability

The data presented in this study are available upon request from the corresponding author.
